# Short Term Synaptic Depression Imposes a Frequency Dependent Filter on Synaptic Information Transfer

**DOI:** 10.1371/journal.pcbi.1002557

**Published:** 2012-06-21

**Authors:** Robert Rosenbaum, Jonathan Rubin, Brent Doiron

**Affiliations:** 1 Mathematics, University of Pittsburgh, Pittsburgh, Pennsylvania, United States of America; 2 Center for the Neural Basis of Cognition, Pittsburgh, Pennsylvania, United States of America; Indiana University, United States of America

## Abstract

Depletion of synaptic neurotransmitter vesicles induces a form of short term depression in synapses throughout the nervous system. This plasticity affects how synapses filter presynaptic spike trains. The filtering properties of short term depression are often studied using a deterministic synapse model that predicts the mean synaptic response to a presynaptic spike train, but ignores variability introduced by the probabilistic nature of vesicle release and stochasticity in synaptic recovery time. We show that this additional variability has important consequences for the synaptic filtering of presynaptic information. In particular, a synapse model with stochastic vesicle dynamics suppresses information encoded at lower frequencies more than information encoded at higher frequencies, while a model that ignores this stochasticity transfers information encoded at any frequency equally well. This distinction between the two models persists even when large numbers of synaptic contacts are considered. Our study provides strong evidence that the stochastic nature neurotransmitter vesicle dynamics must be considered when analyzing the information flow across a synapse.

## Author Summary

Neurons communicate through electro-chemical connections called synapses. Action potentials in a presynaptic neuron cause neurotransmitter vesicles to release their contents which then bind to nearby receptors on a postsynaptic neuron's membrane, transiently altering its conductance. After it is released, the replacement of a neurotransmitter vesicle takes time and the depletion of vesicles can prevent subsequent action potentials from eliciting a postsynaptic response, an effect that represents a form of short term synaptic depression. When a vesicle is available for release, an action potential elicits its release probabilistically and depleted vesicles are replenished randomly in time, making the transmission of presynaptic signals inherently unreliable. We analyze a mathematical model of vesicle release and recovery to understand how signals encoded in sequences of presynaptic action potentials are reflected in the fluctuations of a postsynaptic neuron's conductance. We find that slow modulations in the rate of presynaptic action potentials are more difficult for a postsynaptic neuron to detect than faster modulations. This phenomenon is only observed when randomness in vesicle release and replacement is taken into account. Thus, by including stochasticity in the workings of synaptic dynamics we give new qualitative understanding to how information is transferred in the nervous system.

## Introduction

**Figure 1:**
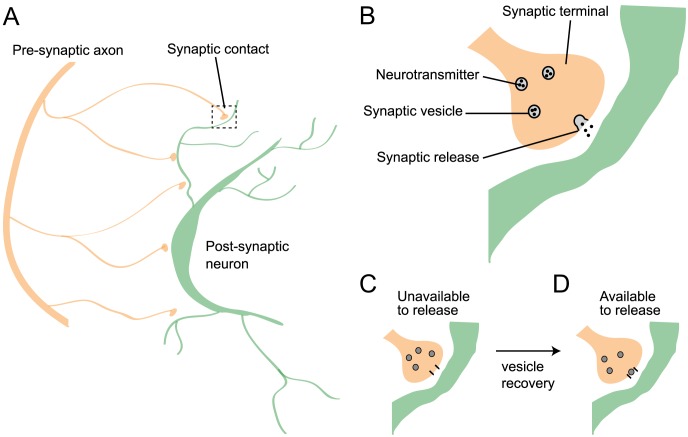
Synaptic vesicle dynamics. (**A**) The axon of a presynaptic neuron (orange) makes 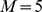 synaptic contacts onto a postsynaptic neuron (green). (**B**) Synaptic vesicles in the synaptic terminal of the presynaptic neuron contain neurotransmitter molecules. A presynaptic action potential releases these neurotransmitter molecules with some probability, 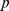. Once released, these molecules bind to the postsynaptic neuron's membrane and cause a transient change in membrane conductance. (**C,D**) After a vesicle is released, the synapse enters a refractory state where it is unavailable to release additional neurotransmitter until it recovers by replacing the released vesicle.

Synapses act as information gates in neuronal networks. Presynaptic action potentials are communicated to postsynaptic neurons by causing synaptic neurotransmitter vesicles to release their contents, which then bind to receptors on a postsynaptic neuron's membrane, evoking a transient change in membrane conductance. After a vesicle is released, it typically takes several hundred milliseconds for it to be replaced at a synaptic contact (see Fig. 1 for a schematic of synaptic release and recovery). This refractoriness induces a form of short term synaptic depression that alters the filtering properties of synapses [1]. An accurate description of synaptic vesicle dynamics and their impact of on information transfer is necessary for a thorough understanding of coding in neuronal networks.

A widely used model of synaptic depression treats vesicle release and recovery as deterministic processes [2]–[6]. While this deterministic model accurately describes the trial-averaged synaptic response to a presynaptic spike train presented repeatedly to a cell [7]–[11], it fails to capture the variability introduced at each trial by the probabilistic nature of vesicle release and recovery [12]. Regardless, the model has been used in studies for which neural variability and information transfer are central themes [13]–[18]. The aim of our paper is to determine the impact (if any) of stochastic vesicle dynamics on the filtering properties of depressing synapses.

Past studies have begun to address this aim by considering how variability from stochastic vesicle release and recovery affects the amount of information transmitted through a synapse as well as the firing rate of a postsynaptic cell [12], [19], [20], but a thorough investigation of the impact of stochastic vesicle dynamics on synaptic filtering has not been performed. We derive a compact description of the filters imposed by short term synaptic depression when stochastic vesicle dynamics are taken into account and when they are ignored. We find that variability introduced by stochastic vesicle dynamics plays a fundamental role in shaping the way in which depressing synapses filter presynaptic information. In particular, a model that ignores this variability transmits presynaptic information encoded at any frequency with the same fidelity [16], [17]. In contrast, a model that captures this variability reduces overall information transmission, and transmits quickly varying signals with higher fidelity than slowly varying signals. Differences between the two models persist over a broad range of physiologically motivated parameter values, even when a large number of synaptic contacts is considered and even at the population level. Our results suggest important implications for how signals encoded at different timescales are propagated through the nervous system and show that synaptic variability must be taken into account to accurately address such questions.

## Results

**Figure 2:**
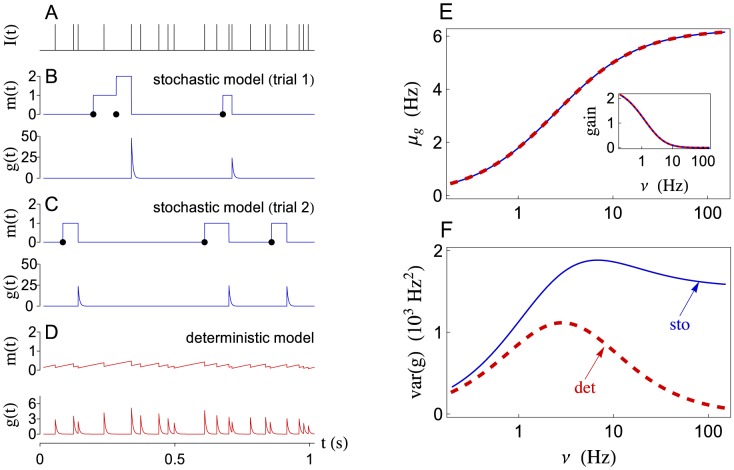
Stochastic versus deterministic models of short term depression. (**A**) An example presynaptic spike train, 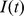. Each vertical bar represents an action potential. (**B**) The number of synaptic vesicles, 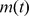, available for release and the conductance, 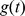, induced in the postsynaptic cell for one realization of the stochastic model. Filled circles in (B) represent vesicle recovery events. (**C**) A second realization of the stochastic model with the same input. Observe in (B) and (C) that the number of vesicles released by the stochastic model during one second is primarily determined by the number of recovery events during that second and does not reflect the number of presynaptic spikes. (**D**) The number of synaptic vesicles and the conductance induced by the deterministic model with the input from (A). Parameters in (A–D) were chosen for illustrative purposes as 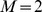, 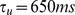, 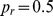, and 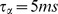. (**E**) The steady state mean conductance, 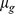, as a function of the presynaptic firing rate, 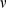. The inset shows the gain, 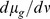. (**F**) The steady state variance of 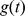 as a function of 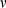 for the deterministic (solid blue) and stochastic (dashed red) models of vesicle dynamics with Poisson inputs. Variability in the deterministic model is introduced only by variability in the input, 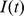. Synaptic parameters for (E–F) and for all subsequent figures are given in Table 1.

We study the synaptic filter induced by short term depression with both a stochastic model and a deterministic model of synaptic vesicle dynamics (see Fig. 2A–D for an illustration and Methods for a detailed discussion). For both models, we consider a presynaptic spike train, 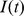, with rate 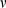 that induces a postsynaptic conductance,
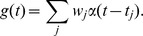
Here, 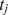 is the time of the 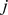
th presynaptic spike, 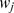 is the number of vesicles released by the 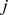
th presynaptic spike, and 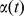 represents the time course of conductance induced by the release of a single synaptic vesicle. The presynaptic cell makes 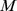 contacts with the postsynaptic cell. We make a simplifying assumption that each contact contains only one release site, so that a single presynaptic action potential can release at most one vesicle per contact [21], hence 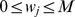. Alternately, to model biological settings where this single vesicle hypothesis is violated [22], [23], 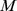 can be interpreted as the total number of release sites across all contacts (see Discussion). We rescale conductance units so that 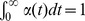. This rescaling causes 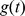 to have dimension 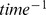 but simplifies the exposition.

In the stochastic model of vesicle dynamics [12], [19], [24], [25], a presynaptic spike releases each available vesicle at each contact independently with probability 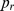. After a contact releases its vesicle, it is unavailable to release again until the vesicle is replaced, a process known as recovery. The waiting time until the vesicle is replaced follows an exponential distribution with mean 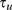 (Fig. 2B,C). For the deterministic model of vesicle dynamics [2], the number of available vesicles is treated as a continuous variable where a proportion 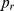 of the total available vesicles are released by each presynaptic spike and the number of available vesicles increases exponentially towards 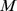 with timescale 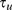 between releases (Fig. 2D). Stochasticity in the conductance, 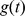, produced by the deterministic model is introduced solely by the stochasticity in the input, 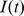. Several presentations of the same realization of 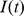 produce the same 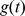 for the deterministic model, but not for the stochastic model (Fig. 2A–D).

The conductance produced by the deterministic model represents the quantity that would be obtained by presenting the same realization of 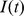 to the stochastic model over several trials, then computing the trial-averaged conductance. Despite the agreement of their trial-averages, though, individual realizations of the two models differ substantially. The deterministic model responds to every presynaptic input, but releases a fractional number of vesicles at each response (Fig. 2D). In contrast, the stochastic model responds to only a few inputs, but releases a larger, quantal number of vesicles at each response (Fig. 2B,C).

The steady state mean conductance induced by a presynaptic spike train 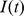 with rate 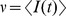 is given by 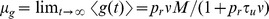 for both the stochastic and deterministic models of vesicle dynamics (Fig. 2E and Eq. (25)). The degree to which a small shift of the presynaptic rate is reflected in a shift of the steady state mean conductance is measured by the gain,
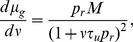
(1)which is a decreasing function that decays to zero as 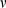 increases, a well-known effect that is due to the saturation of the mean conductance for large presynaptic firing rates (see Fig. 2E, inset and [2], [3], [26]). However, the gain only measures changes in the *steady state* mean of 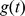 after a *sustained* shift in the mean of 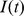, whereas the signal processing properties of a synapse also depend on the *temporal* response of 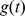 to *transient* fluctuations in 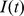 [3], [10], [27], [28]. Below, we use a cross-spectral measure to quantify the temporal response properties of 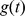.

The information processing capabilities of a synapse depend not only on the response of 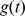 to temporal fluctuations in 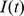, but also on the temporal and trial-to-trial variability of 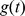. Noise introduced by stochastic vesicle release and recovery leads to larger variability in 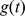, as measured by its variance (Fig. 2F). However, the variance alone does not capture the timescale over which this variability occurs. Below, we use a power-spectral measure to describe the variability of 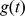 over different timescales.

### Synaptic filtering of a Poisson presynaptic spike train

To gain an intuition for the signal processing properties of depressing synapses, we first study the case of a single Poisson presynaptic spike train, 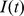, with constant rate 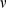. Since a homogeneous Poisson process has equal power at every frequency, this approach allows us to investigate synaptic filtering at all frequencies simultaneously. Later, we will consider the response to an inhomogeneous Poisson process whose rate encodes a signal.

**Figure 3:**
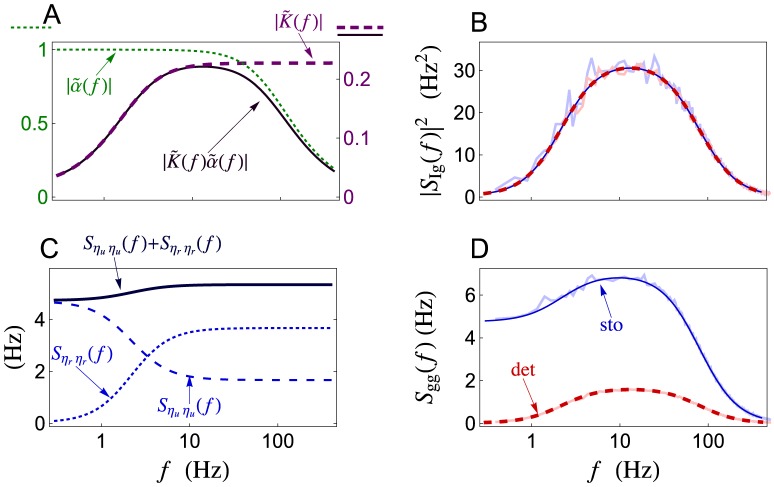
Synaptic filtering of a single Poisson presynaptic spike train. (**A**)**–**(**B**) The low-pass filter, 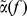, and the high-pass filter, 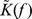, are multiplied with the presynaptic rate (*cf.* Eq. (2)) to determine the band-pass cross-spectrum, 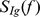, between a Poisson presynaptic spike train, 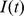, and postsynaptic conductance, 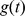. The cross-spectrum is identical for the stochastic (solid blue) and deterministic (dashed red) models. (**C**)**–**(**D**) The power spectrum, 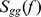, of the conductance is larger for the stochastic model than the deterministic model due to the additive terms, 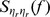 and 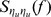, that quantify the increase in variability due to stochastic vesicle release and recovery (see Eq. (3)). For this and all subsequent figures, solid blue lines and dashed red lines show plots obtained from closed form expressions for the stochastic and deterministic models, respectively. Light blue and light red lines indicate simulations of the stochastic and deterministic models, respectively.

The magnitude of the response of the conductance, 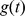, at frequency 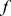 to fluctuations in the input, 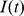, is quantified by the cross-spectrum, 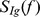, between these quantities (see Methods). For both the deterministic and stochastic models of vesicle dynamics, the cross-spectrum is given by (see Eq. (25) in Methods)
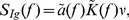
(2)where 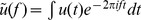 denotes the Fourier transform and 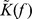 is a kernel that captures the filtering properties of synaptic depression (see Eq. (20) in Methods and Fig. 3A). The fact that 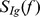 is identical for the stochastic and deterministic models can be understood intuitively by noting that stochasticity in vesicle dynamics is uncorrelated from 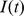 and therefore does not contribute to the covariability of 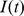 and 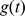. It should be noted that, while Eq. (2) is exact for the deterministic model, it is an approximation for the stochastic model (see Methods), which is validated by simulations (Fig. 3B).

The shape of 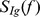 can be understood by its components in Eq. (2). The low-pass filter, 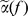, which captures postsynaptic channel dynamics, suppresses power at frequencies higher than 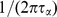 (see Fig. 3A and [29]). The high-pass filter 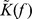, which captures the deterministic dynamics of short term depression, suppresses power at frequencies lower than 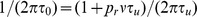 (see Fig. 3A, Methods and [17]). Their product, which determines 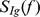 through Eq. (2), is then band-pass with most of its power at frequencies between 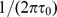 and 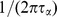 (Fig. 3B). Thus, only fluctuations in the presynaptic input within this frequency band are reflected faithfully by fluctuations in the postsynaptic conductance.

**Figure 4:**
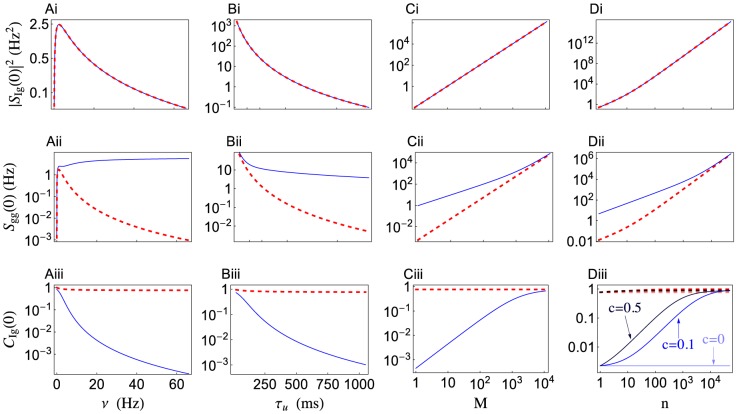
Low frequency signal transfer in a variety of parameter regimes. Low frequency cross-spectrum (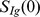), auto-spectrum (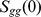), and coherence (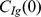) between a Poisson presynaptic spike train, 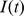, and postsynaptic conductance, 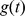, plotted as a function of the presynaptic rate, 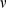 (**Ai–iii**), the vesicle recovery timescale, 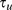 (**Bi–iii**), the number of synaptic contacts, 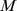 (**Ci–iii**), and presynaptic population size, 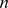 (**Di–iii**). Columns A–C are for a single presynaptic spike train (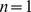). The zero-frequency coherence in Diii is shown for three values of the presynaptic correlation coefficient: 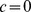, 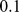, and 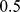. The power spectrum and coherence predicted by the stochastic model (solid blue) and the deterministic model (dashed red) disagree by orders of magnitude unless 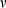 is small, 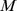 is large, 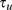 is small, or 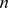 is large with 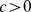.

The low-frequency limit of 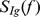 is nearly zero for the parameter values chosen in Table 1 (Fig. 3B). This can be explained by noting that the zero-frequency cross-spectrum is related to the gain by [30]

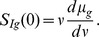
For large 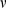, the mean conductance saturates and the gain decays to zero like 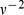 (see Eq. (1) and Fig. 2E). Thus, 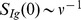 which decays to zero for large 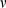 (Fig. 4Ai). More specifically, 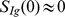 when vesicles become depleted, which occurs when release is faster than recovery, i.e., 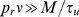. Note, though, that 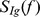 is larger for higher frequencies, meaning that faster fluctuations in 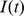 cause larger transient fluctuations in 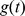 when compared to changes in the steady state mean conductance, 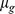, caused by static changes in 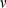 [3], [10], [27], [28].

**Table 1:** Table of synaptic parameters.

Name	Definition	Default value
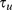	timescale of vesicle recovery	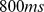
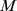	number of contacts between a pre- and postsynaptic cell	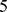
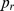	probability of release when vesicle is available	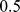
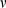	presynaptic rate	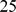 Hz
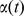	synaptic activation kernel	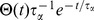
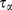	time constant of postsynaptic channels	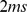
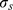	bandwidth of rate-coded signal	0.1 Hz
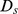	peak power of rate-coded signal	20 Hz
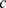	noise correlation between presynaptic spike trains	0.1

Parameters for synapses and presynaptic spike trains. These parameter values are used in all figures unless otherwise indicated. Here, 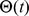 represents the Heaviside step function.

The trial-to-trial and temporal variability of the conductance at frequency 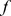 is quantified by its power spectrum, 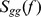, which is given by (see Eq. (25) in Methods)
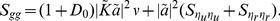
(3)Here 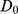 is a constant that represents variability introduced by the interaction of Poisson input with deterministic vesicle dynamics, 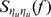 captures variability introduced by stochastic recovery, and 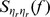 captures variability introduced by probabilistic vesicle release. For the deterministic model, 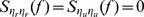, but 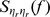 and 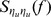 are positive for the stochastic model (see Methods and Fig. 3C). As a result, the stochastic model predicts a larger power spectrum than the deterministic model (Fig. 3D). The decay of 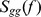 at high frequencies is due to the low-pass nature of the synaptic conductance kernel, 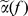 (see Fig. 3A and [29]).

The power spectrum predicted by the two models differs most significantly at low frequencies, where it is nearly zero for the deterministic model but much larger for the stochastic model (Fig. 3D). This can be understood by noting that [30]

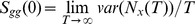
where 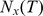 is the number of vesicles released in a window of length 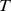. For the parameter values in Table 1, 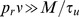 so that vesicles are mostly depleted and therefore the number of vesicles released in a large time window is determined largely by the number of recovery events during that window (Fig. 2A–D). For the stochastic model, recovery events at each contact occur as a Poisson process with rate 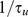. Since there are 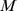 contacts and a Poisson process has power equal to its rate, 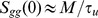 when 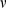 is large. This intuition is confirmed by noting that 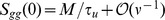 for the stochastic model. In contrast, for the deterministic model, recovery is deterministic and therefore the amount of neurotransmitter taken up, and hence released, over a large time window has a small variance. This is confirmed by noting that 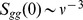 for the deterministic model and therefore approaches zero for large 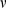. For the synaptic parameters in Table 1, the power spectra produced by the stochastic and deterministic models disagree for 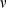 larger than a few Hz (Fig. 4Aii).

**Figure 5:**
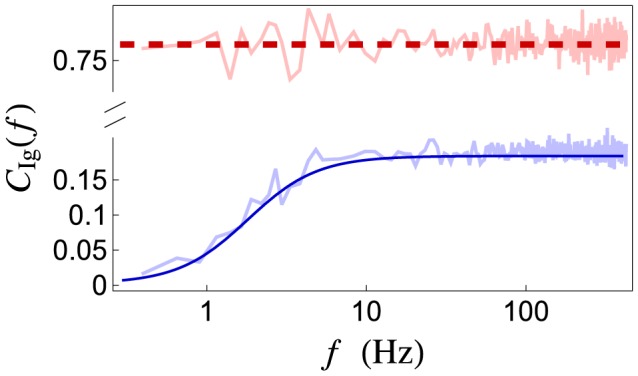
Coherence between a single presynaptic spike train and the postsynaptic conductance it induces. The coherence, 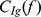, between a Poisson presynaptic spike train, 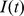, and the resulting postsynaptic conductance, 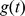. The stochastic model (solid blue) yields a high pass coherence that is dramatically smaller than the flat coherence predicted by the deterministic model (dashed red).

The fidelity with which fluctuations in the postsynaptic conductance, 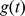, reflect fluctuations of the input, 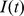, at frequency 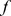 is quantified by their coherence
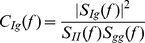
where 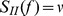 is the power spectrum of the Poisson input. Since 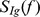 is identical for the two models, but 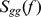 is larger for the stochastic model (Fig. 3B,D), it follows that 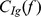 is smaller for the stochastic model (Fig. 5). We now investigate the differences between the coherences produced by the two models in more depth.

Since 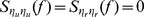 for the deterministic model, the cross-spectrum, 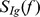, and power spectrum, 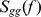, are proportional to one another (see Eqs. (2) and (3)) so that dividing them gives a flat coherence (i.e., a coherence that does not depend on 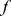, Fig. 5 and [16], [17]),
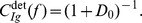
Here and in subsequent expressions, a 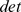 (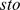) superscript indicates identities for the deterministic (stochastic) model. Synaptic variability in the stochastic model increases the power spectrum, giving a frequency-dependent coherence
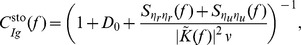
which is high-pass (Fig. 5). Thus, stochastic vesicle dynamics introduce high-pass frequency dependence into the fidelity of a synaptic filter.

In addition to introducing frequency dependence, stochastic vesicle dynamics also decrease the coherence substantially, especially at lower frequencies where the coherence is nearly zero for the stochastic model (Fig. 5). The fact that coherence is small at low frequencies for the stochastic model can be understood intuitively through the following relation [30],
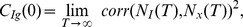
where 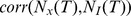 is the Pearson correlation coefficient between the number of presynaptic spikes, 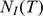, and the number of vesicles released, 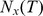, in a window of length 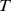. When 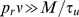, synapses are mostly depleted in the steady state. As a result, the number of vesicles released during a long time interval is determined primarily by the number of recovery events in that time window and hence mostly independent of the number of presynaptic spikes (Fig. 2A–C and [31]). Therefore, for the stochastic model, the number of vesicles released over a long time window is uncorrelated from the number of presynaptic spikes and, as a result, 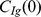 is small.

These intuitions are confirmed by appealing to the asymptotic expressions derived for the cross-spectrum and power spectrum above. For the stochastic model, 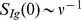 and 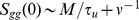 when 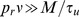. Since 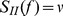 for Poisson input, it is then clear that
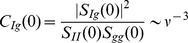
for the stochastic model when 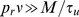. For the deterministic model, however, 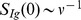, 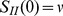, and 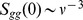 so that 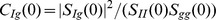 approaches a positive constant for 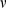 sufficiently larger than 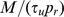. For the parameter values in Table 1, the coherences for the stochastic and deterministic models disagree substantially when 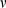 is more than a few Hz (Fig. 4Aiii).

The disagreement between the stochastic and deterministic models is most dramatic when 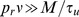 since the postsynaptic response is determined primarily by vesicle recovery dynamics in this regime, as discussed above. In the figures considered so far, we have used 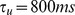, motivated by measurements of pyramidal–to–pyramidal synapses in rodent neocortex [2], [19]. However, both shorter and longer time constants have also been reported in cortex [5], [7], [8], [32], [33]. When other parameters are set to the values from Table 1, the two models disagree substantially when 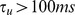 (see Fig. 4Bi–iii).

A proposed justification for using a deterministic model of vesicle dynamics is that stochasticity introduced at each contact averages out when a presynaptic cell makes several contacts [17]. The number, 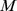, of contacts a presynaptic cell makes with a single postsynaptic cell varies greatly across cell subtypes and brain regions. Rodent and cat pyramidal cells in the hippocampus and neocortex typically make 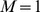
–12 contacts onto other pyramidal cells or onto interneurons. Interneurons in the same regions make 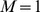
–17 contacts onto pyramidal cells. On the other hand, the Calyx of Held synapse can make more than 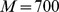 contacts onto a single postsynaptic target in the rodent auditory brainstem and Purkinje cells can receive over 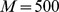 contacts from single presynaptic cells in the rodent cerebellum (see [34] for values of 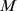 measured in various animals and synapses). When other parameters are set to the values from Table 1, the stochastic and deterministic models disagree substantially for 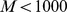 (see Fig. 4Ci–iii).

In summary, over a broad range of synaptic parameters, stochastic vesicle dynamics both attenuate and impart a high-pass nature to the coherence between a pre-synaptic spike train and the post-synaptic conductance response. We next explore the implications of these effects on the transfer of rate-coded information.

### Synaptic filtering of a rate-coded signal

Time-varying stimuli are often encoded in fluctuations of the firing rate of neuronal populations [35]. To address the question of how information about a rate-coded signal is filtered by vesicle dynamics, we use a model from [16] and [17] in which a time-varying signal is encoded in the firing rate of a presynaptic spike train to yield a doubly stochastic Poisson process, 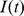 (see Methods).

In this model, the instantaneous presynaptic rate conditioned on a signal, 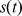, is given by 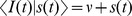 and, without conditioning on 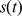, is given by 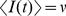. The power spectrum of the presynaptic spike train is given by
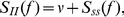
(4)where 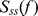 is the power spectrum of 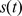. Eq. (4) can be interpreted as follows: 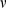 represents the power of Poisson noise and 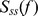 represents the power of the signal. Unless 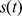 is identically zero, 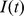 inherits non-Poisson statistics from 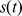, which violates the Poisson assumptions used to derive the spectral properties given above. In the Methods, we derive a linear approximation (valid when 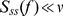) to the synaptic filter induced by the deterministic and stochastic models of vesicle dynamics and use it to obtain approximations to the cross-spectrum, 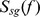, between the signal and conductance as well as the power spectrum, 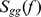, of the conductance for this model (see Eqs. (27) and (28) in the Methods). These approximations allow an investigation of the information transfer of the signal across the synapse in various frequency bands.

**Figure 6:**
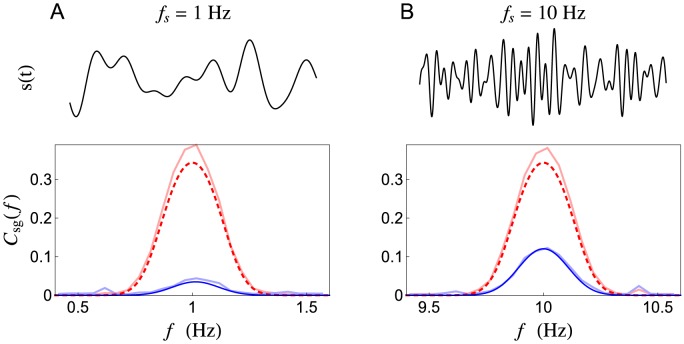
Signal transfer at high and low frequencies. The firing rate of a single presynaptic spike train (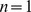) is modulated by the signal, 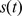, producing a postsynaptic conductance, 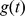. The coherence between the signal and conductance for (**A**) a slowly varying signal with peak frequency 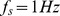 and (**B**) a quickly varying signal with 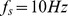. The stochastic model (solid blue) transmits the higher frequency signal more reliably than the lower frequency signal. The deterministic model (dashed red) transmits the signal with equal fidelity in both cases.

We model 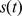 as a Gaussian process with Gaussian-shaped power spectrum (Fig. 6A,B),
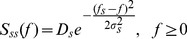
(5)where 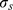 is the bandwidth, 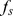 the central frequency, and 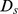 the peak power of the signal. We use a narrow-band signal (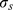 small) to more clearly illustrate the dependence of synaptic fidelity on signal frequency. Since 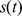 is Gaussian, there is a positive probability that 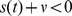 so that the instantaneous firing rate of the presynaptic cells becomes negative. However, when 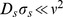, this occurs rarely and can be disregarded by considering negative rates as zero [17]. The coherence, 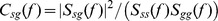, between the signal and the conductance quantifies the fidelity with which the signal, 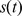, is represented in the postsynaptic conductance, 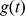. For the deterministic model of vesicle dynamics, the coherence is given by (from Eqs. (27))
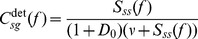
so that changing 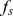 merely shifts 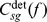, but does not change its amplitude (Fig. 6C,D dashed red line). Thus, a signal coded within any frequency band is transmitted with the same fidelity, consistent with the conclusions reached above using the Poisson model and also consistent with previous studies [16], [17]. For the stochastic model, however,
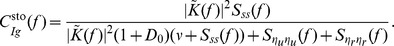
Since 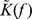 is high pass (Fig. 3A) and 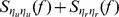 is mostly flat (Fig. 3B), 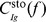 is larger when 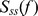 concentrates its power in higher frequencies. For example, the amplitude of the coherence is larger when 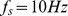 than when 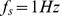 for the stochastic model, but independent of 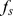 for the deterministic model (Fig. 6C,D).

**Figure 7:**
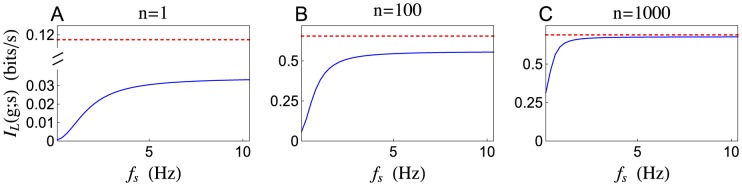
Linear information transfer rate as a function of signal frequency. The linear mutual information rate, 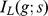, between a rate-coded signal, 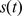, and the total conductance, 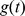, produced by (**A**) 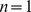, (**B**) n = 100, and (**C**) 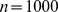 presynaptic spike trains, each encoding 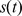. The information rate is plotted as a function of the central frequency, 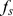, at which 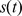 is encoded. The stochastic model (solid blue) transmits quickly varying signals more reliable than slowly varying signals. The deterministic model (dashed red) transmits information encoded at any frequency equally well.

The rate of linear information transferred from the signal to the conductance is given by [36], [37]

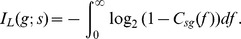
In particular, 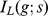 represents the total information per unit time that a linear decoder can obtain about the signal, 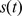, by observing the conductance, 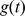, and also represents a lower bound on the Shannon information [36], [37]. The stochastic model predicts a dramatically lower linear information rate than the deterministic model (Fig. 7A). Since, for the deterministic model, the amplitude of 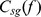 is independent of the central signal frequency, 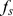, the linear information rate is also independent of the central frequency (Fig. 7A). The stochastic model, however, transmits quickly varying signals with more fidelity than slowly varying signals (Fig. 7A). Hence, stochastic vesicle dynamics introduce frequency dependence into the transfer of linear information across a synapse.

In summary, our results show that the high pass nature of synaptic depression combined with low frequency synaptic noise limits the transfer of low frequency information through a synapse, while higher frequency information is transmitted more reliably. We next investigate these conclusions in a population setting.

### Synaptic filtering at the population level

So far, we have studied the conductance induced by a single presynaptic spike train that makes several contacts onto a postsynaptic cell. However, information about a stimulus is often encoded by populations of several presynaptic cells. We now consider a population model in which a collection, 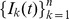, of 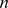 presynaptic spike trains all encode the same signal, 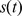, as described for the single-cell model above. These inputs induce individual synaptic conductances, 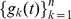, in a single postsynaptic cell. Define the total presynaptic input, 
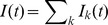
, and the conductance induced by this input, 
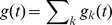
. For simplicity, we assume that all synapses have the same synaptic parameters 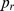, 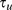, 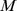, and 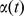.

The signal, 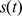, introduces variability that is shared between the presynaptic spike trains. Such shared variability is commonly referred to as *signal correlation* since it is informative of the signal. Populations of presynaptic neurons that code for the same stimulus also share non-informative variability, known as *noise correlation* [38], [39]. As a simple model of presynaptic noise correlation, we assume that each pair of spike trains, 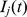 and 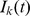 with 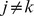, share a proportion 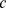 of their spike times. The pairwise cross-spectra are then given by
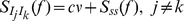
where 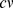 represents the contribution of noise correlations and 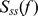 represents the contribution of signal correlations.

**Figure 8:**
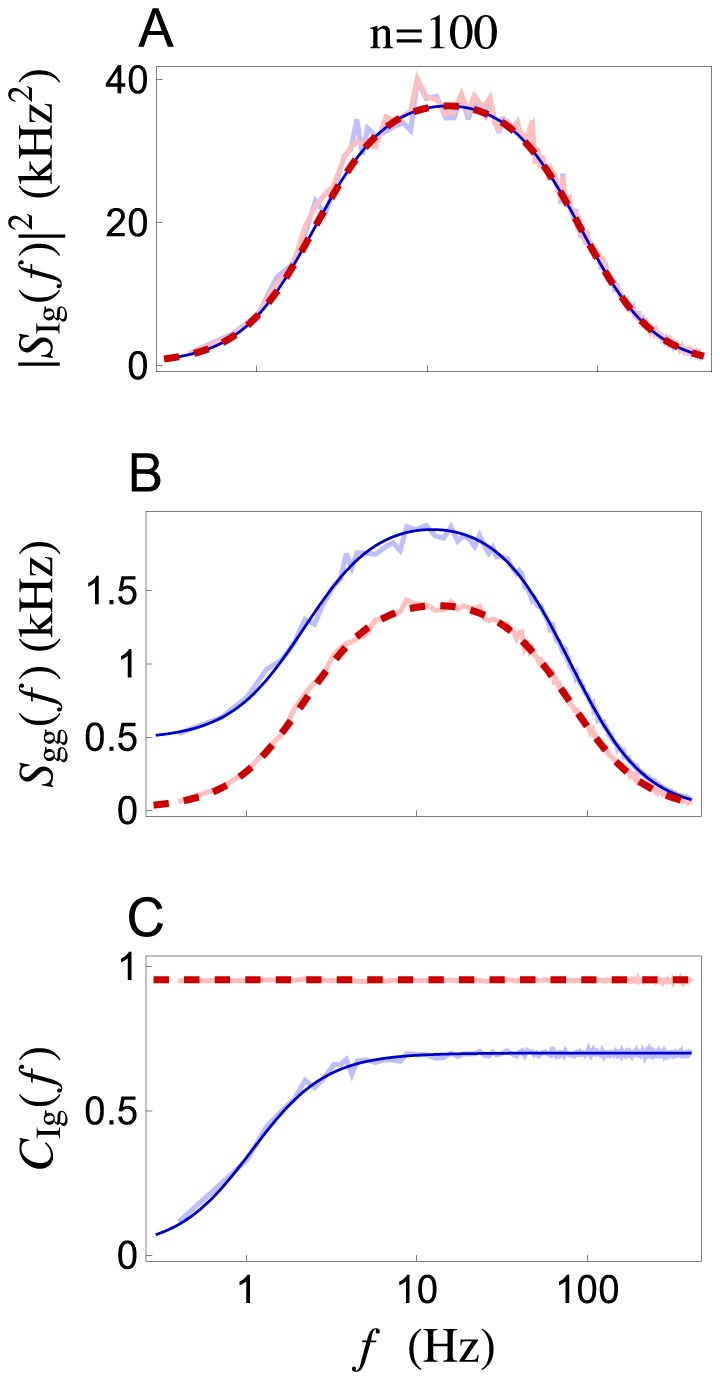
Synaptic filtering at the population level. A population, 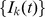, of 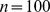 Poisson presynaptic spike trains with pairwise correlation 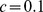 drive a postsynaptic neuron to produce postsynaptic conductances, 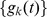. (**A**) The cross-spectrum between the total presynaptic input and the total conductance. (**B**) The power spectrum of the total conductance has maximal power within the beta frequency band for both the deterministic (dashed red) and stochastic (solid blue) models. (**C**) The coherence between the total presynaptic input and the total conductance. Stochastic vesicle dynamics increase the power spectrum and therefore decrease the coherence, especially at low frequencies. All three plots are obtained in the absence of a rate-coded signal (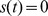).

As we have done for the single input model above, we gain an intuition for the population-level filter imposed by short term depression by first considering purely Poisson spike trains, which is achieved by setting 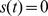 so that 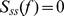. Even though the cross-spectrum, 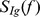, is identical for the stochastic and deterministic models, the power spectrum, 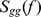, is larger for the stochastic model due to noise introduced by synaptic variability (see Fig. 8A,B and Eq. (29) in Methods). Therefore the coherence, 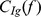, between the total presynaptic signal and the total conductance is smaller for the stochastic model. Moreover, the deterministic model predicts a flat coherence, while the stochastic model predicts a high-pass coherence (Fig. 8C). These conclusions are identical to those reached for a single input above, but the disparity between the two models is reduced at the population level (compare Figs. 3 and 5 with Fig. 8).

Notice also that the power spectrum, 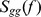, is peaked within the beta frequency band even though the inputs are Poisson and therefore have a flat power spectrum. This effect could exaggerate beta frequencies in recorded data. We return to this topic in the Discussion.

A potential justification for using a deterministic model of vesicle dynamics is that, since stochastic release and recovery events are uncorrelated across all synapses, the extra variability introduced by synaptic noise averages out at the population level. So far, we have compared the two models for a population size of 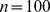. For the parameter values in Table 1, the low frequency cross-spectrum is identical for the two models, but the coherence and power spectrum disagree considerably until 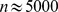 (Fig. 4Di–iii). The value of 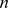 at which the models begin to agree depends on the pairwise correlation, 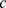, between the presynaptic inputs. Notably, in the absence of correlations (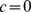 and 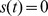), the population-level coherence is identical to the individual coherences, 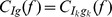, so that the coherence predicted by the stochastic and deterministic models disagree by the same amount for any value of 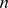 (Fig. 4Diii, lightest lines). As 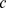 increases, the two models agree at smaller population sizes (Fig. 4Diii, darker lines). Hence, presynaptic correlations must be present and 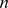 must be large if the deterministic model is to be used in place of the stochastic model for large populations.

We now study the transfer of rate coded information at the population level by allowing 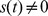. In particular, we are interested in how information about a rate-coded signal, 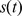, is transferred to the population conductance, 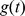. As above, we use a signal with Gaussian shaped power spectrum given by Eq. (5). A linear approximation to the cross-spectrum, 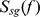, for this model is calculated in the Methods (see Eq. (29)), which allows us to calculate the coherence, 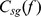, between the signal and the postsynaptic response and the linear information rate, 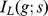, which depends on the central frequency at which the signal is coded in a qualitatively similar manner as for a single presynaptic spike train (compare Figs. 7A to 7B,C). In particular, low frequency information transfer is reduced for the stochastic model of synaptic depression. Moreover, the stochastic model transfers information in a frequency dependent manner and the deterministic model transfers information at all frequencies equally (Fig. 7). The disparity between the models is substantial when 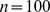, but reduced considerably when 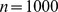 (compare panels B and C in Fig. 7). We remind the reader that 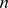 represents the number of presynaptic neurons that encode the shared signal, 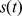, which could be much smaller than the total number of presynaptic inputs a cell receives. This suggests that, due to the stochastic nature of vesicle release and recovery, large presynaptic populations must be used to encode slowly varying signals.

## Discussion

We derived a concise mathematical description of the synaptic filter induced by short term depression arising from neurotransmitter vesicle depletion. We found that stochasticity in vesicle release and recovery plays an important role in shaping this filter and determining the information processing capabilities of depressing synapses. For example, ignoring the stochasticity introduced by stochastic vesicle dynamics gives rise to a filter that transmits rate-coded signals encoded at all frequencies equally well [16], [17], but taking this stochasticity into account reduces information transfer and causes slowly varying signals to be transferred with higher fidelity than slowly varying signals.

The deterministic model of short term depression provides a usable approximation to the stochastic model when considering large populations of correlated presynaptic spike trains (Figs. 4Di–iii and 7C). While a postsynaptic neuron typically receives thousands of inputs, only a fraction of these inputs might be devoted to encoding a single stimulus. Our results show that a slowly varying stimulus must be encoded by large presynaptic populations, but quickly varying stimuli can be encoded by smaller populations. This conclusion is not true the deterministic model of synaptic depression, which ignores the inherent randomness of vesicle dynamics.

Since the two models predict the same mean conductance, the deterministic model is valid for studies that focus on mean postsynaptic activity and for which noise is not a concern. For example, the deterministic model has been used to describe the effects of depression on gain and temporal changes in postsynaptic firing rate [3], [10], [26], [27]. Using the deterministic model in these cases is justified only if changes in postsynaptic firing rate result primarily from changes in the mean conductance and the variability of the conductance is inconsequential. When spiking is fluctuation driven, the postsynaptic firing rate is underestimated by the deterministic model [12].

A number of experimental studies have successfully fit parameters for the deterministic model to recorded neural data. This is achieved by first repeating the same presynaptic stimulus to a cell, then averaging the cell's response and fitting the averaged response to the response predicted by the deterministic model [2], [5], [7], [8], [18], [32], [33]. Since the stochastic model discussed here uses the same parameters as the deterministic model, the parameters obtained through this procedure can also be used to constrain the stochastic model.

### Spectral analysis of synaptic depression

There is an extensive experimental and theoretical literature addressing how synapses that exhibit short term depression transmit different patterns of presynaptic spikes [3], [26], [27], [40], [41]. One recurring observation in these studies is that the steady state mean conductance (equivalently, the mean rate of vesicle release) saturates with the presynaptic firing rate, which causes the gain, 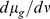, to approach zero for large presynaptic rates (Fig. 2E). However, the gain only captures the sensitivity of the steady-state mean, 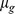, to static changes in 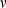. Previous studies show that temporal changes in 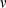 are reflected more reliably in the transient mean of 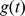 than static changes of 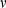 are reflected in the steady-state mean of 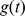 [3], [10], [27], [28]. This observation can be understood through our analysis by noting that higher frequency components of 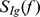 are larger than the low-frequency components (Fig. 3B). Note that the decay of 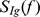 at very high frequencies is due to the low-pass properties of the post-synaptic conductance kernel, 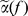, (Fig. 3A and [29]) and not to synaptic depression. The filtering effects of depression are captured by the kernel 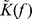, which is high-pass (Fig. 3A).

A second shortcoming of the gain as a descriptive quantity is that it does not capture the trial-to-trial variability in the conductance, which is a vital component of information transfer. We quantify this trial-to-trial variability as a function of frequency using the power spectrum, 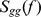. We show that the frequency-independence of information transfer through a deterministic synapse model depends on the precise shape of 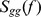 [16], [17], and the high-pass frequency-dependence of information transfer through a stochastic synapse model likewise depends on the shape of 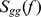. Furthermore, we show that stochastic vesicle dynamics cause an overall decrease in information transfer by increasing 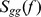. Thus, trial-to-trial variability in 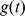 must be considered to obtain an accurate description of information transfer through a synapse.

While other studies of synaptic depression have investigated the transfer of rate-coded signals at various frequencies, we are not aware of a study that derives an explicit approximation to the filter induced by a depressing synapse. Such an approximation is derived in the Methods, giving
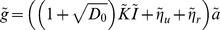
where 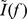 and 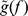 are the Fourier transforms of the presynaptic spike train and postsynaptic conductance respectively (see Methods for definitions of other terms). This expression can be used to predict the spectral properties of the postsynaptic response to a presynaptic input with a given power spectrum. A generalization of this expression that can be used in the case of a population of correlated presynaptic spike trains is given by Eq. (26).

### Synaptic depression and neural rhythms

For the parameters in Table 1, the power spectrum is peaked within the beta frequency band (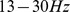) for both the stochastic and deterministic models (Fig. 8B). We emphasize that the presynaptic spike trains in this case are Poisson processes with flat power spectra and cross-spectra. Thus, the peaked power spectrum of the conductance is due completely to synaptic filtering: Frequencies below 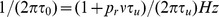 are suppressed by synaptic depression and frequencies above 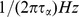 are suppressed by post-synaptic channel dynamics. The conductance power spectrum is peaked between these two frequencies. This effect could potentially cause an exaggeration of beta or other frequencies in recordings such as local field potentials that reflect large pools of synaptic currents. Parameters can be chosen within a physiologically realistic range to produce a more exaggerated peak than that shown in Fig. 8B or to produce a peak within another frequency band (not shown). Further work is needed to determine the role that synaptic filtering plays in generating or exaggerating rhythms within beta or other frequency bands in functioning neural circuits.

### Possible extensions

We used a simplified model of neurotransmitter release and recovery. In particular, we assumed that each contact contains only one release site. However, individual contacts can have multiple release sites and recent results show that multiple vesicles can be released by a single contact in response to a single presynaptic action potential [22], [23]. Such situations can be modeled in our framework by interpreting 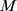 as the total number of release sites at all contacts. However, this interpretation is only valid if the release of vesicles is statistically independent between release sites that share a contact. If the probability of release at one site depends on release at another site – for instance if a contact has several release sites but can only release one vesicle per presynaptic spike [12], [42] – then our model would need to be adjusted to account for this dependency. To the authors' knowledge, the precise structure of such dependencies are a subject of current research and not presently understood. In the depleted state (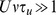), a contact with several release sites will rarely have more than one vesicle available for release at any point in time and our single-vesicle model should provide an accurate approximation regardless of dependencies between release sites, as long as the recovery time constant is properly adjusted [12].

We modeled stochasticity introduced by probabilistic vesicle release and random recovery times, but did not model stochasticity introduced by randomness in the amount of neurotransmitter contained in each vesicle [43], [44]. In addition we did not model variability at the postsynaptic site (e.g., randomness in the number of bound receptors, the number of open channels, or the availability of messenger molecules), which could introduce variability in the amplitude of the postsynaptic conductance elicited by each vesicle released. Assuming statistical independence of these sources of variability between release events, they can be captured by multiplying each response amplitude, 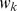, by a random number. This would simply scale the power spectrum of the conductance linearly and would not alter our central conclusions.

The cross-spectrum between presynaptic input and postsynaptic conductance decays to zero at high frequencies, but the coherence between the two does not (Figs. 3A and 5). This is due to the fact that the power spectrum also decays at high frequencies and cancels perfectly with the cross-spectrum. However, any additional high frequency noise would destroy this balance. For example, if one were to instead compute the coherence between the presynaptic input and the *current* across the postsynaptic membrane, high frequency channel noise [45] could increase the power spectrum without increasing the cross-spectrum and therefore cause the coherence to decay at high frequencies. Thus, information transfer from presynaptic input to postsynaptic current is effectively bandpass. Similar observations were discussed in [17] for the deterministic model of vesicle dynamics with additive noise.

We used a linear approximation to predict the spectral properties of the postsynaptic conductance induced by non-Poisson presynaptic spike trains. However, the approximation is only assured to be accurate when inputs are approximately Poisson, i.e., have a nearly flat power spectrum. This restriction is implicit in our assumption that 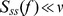 (see Eq. (4) and the surrounding discussion). Presynaptic spike trains that exhibit highly non-Poisson properties, such as bursts or a high degree of regularity, can interact with synaptic depression in a fundamentally different manner than Poisson spike trains [12], [46]. Further work is needed to extend our results to highly non-Poisson presynaptic spiking statistics.

We focused on short term depression caused by the depletion of synaptic neurotransmitter vesicles. However, other sources of short term depression as well as several forms of short term facilitation affect the filtering properties of synapses [1], [40]. Our mathematical methods could be extended to take these additional forms of plasticity into account.

### Synaptic transmission of Shannon information

To quantify information transfer through a synapse, we used an information metric that only captures the amount of information available to a linear decoder observing the conductance. The Shannon information measures the maximum amount of information available to any decoder [47]. Interestingly, for our choice of 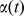, the deterministic model of vesicle dynamics transmits Shannon information perfectly because every presynaptic spike elicits a postsynaptic response (Fig. 2D) and hence each spike time can be resolved by detecting jumps in 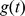 [17], [19]. In contrast, the stochastic model of vesicle dynamics exhibits failures due both to probabilistic release and to vesicle depletion (Fig. 2C,E). Due to the presence of synaptic failure, the stochastic model reduces Shannon information since some presynaptic spikes have no effect on the postsynaptic conductance.

A few studies have investigated the reduction of Shannon information through synapses with synaptic failure [20], [46], [48] but focus on the impact of probabilistic release and ignore stochasticity in vesicle recovery dynamics. In contrast, we studied the reduction of linear information induced by both probabilistic release and stochastic recovery. The qualitative differences we observed between stochastic and deterministic models depend on the stochasticity of vesicle recovery since it introduces low frequency variability into the conductance (Fig. 3C,D). To our knowledge, only one study [19] has investigated information transmission in a model with both probabilistic release and stochastic recovery. Using simulations, they found that stochastic vesicle dynamics reduce Shannon information by orders of magnitude, consistent with our results for linear information. These previous studies of information transmission do not quantify the dependence of information transfer on the frequency band in which presynaptic information is encoded. Furthermore, care must be taken when drawing conclusions about neural coding from studies of Shannon information. Shannon information quantifies the maximal information that can be extracted by a decoder, but it is not always clear whether a neural decoder can achieve optimal or even near-optimal decoding.

## Methods

### Definition of the models and derivation of first moments

Consider a single presynaptic neuron that fires action potentials at times 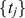 and define the presynaptic spike train as a point process,
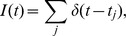
where 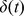 is the Dirac delta function. The number of presynaptic spikes in 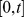 is then given by 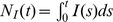. Define 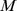 to be the number of functional contacts that the presynaptic neuron makes onto a postsynaptic cell [48] and, for simplicity, assume that each contact can have at most one vesicle available for release at any point in time. Let 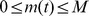 be the total number of vesicles available for release at time 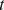. Let 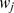 be the number of vesicles released by the 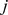
th presynaptic spike, with 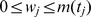. The total number of vesicles released up to time 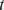 is given by 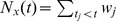 and the effective synaptic input is a marked point process defined by
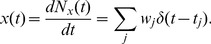
(6)


We first consider a model of synaptic vesicle dynamics that treats vesicle release and recovery stochastically [12], [19], [24], [25]. At each presynaptic spike time, 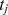, each contact at which a vesicle is available releases this vesicle independently with probability 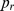. After a synaptic contact releases its vesicle, vesicle recovery occurs as a Poisson process with rate 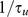. That is, the waiting time from vesicle release until recovery at a single contact is exponentially distributed with mean 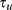 and independent from the state of other contacts, so that the probability of a recovery event during the interval 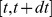 is 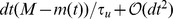. This model can be described by the equation
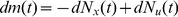
(7)where 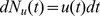 is the increment of an inhomogeneous Poisson process with instantaneous rate that depends on 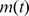 through 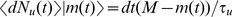 (here, 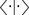 denotes conditional expectation) and 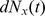 is given by Eq. (6) where each 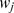 is a binomial random variable with mean 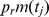. Since each trial with a fixed input, 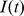, yields a different, random realization of the response, 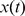, we hereafter refer to this model as the “stochastic model” of vesicle dynamics.

A popular simplification of the stochastic model replaces the random increments, 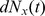 and 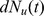, in Eq. (7) with their expected values conditioned on 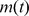 and 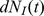 [2], [3], [5], [6]. Since 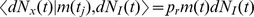 and 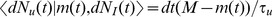, this gives
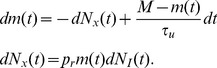
(8)This model treats 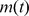 as a continuous variable where a proportion 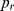 of the available vesicles are released at each input and recovery occurs exponentially with time constant 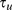. We hereafter refer to the model described by Eq. (8) as the “deterministic model” of vesicle dynamics since the response, 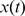, is determined completely by the presynaptic input, 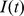. Stochasticity in this model is only introduced by randomness in 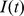.

When 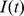 is a homogeneous Poisson process, the deterministic model is analytically tractable: the first two moments of 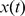 and 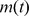 can be derived exactly, as we show below. We also show that the first moments agree for two models. The second moments for the stochastic model are difficult to derive analytically, but we derive a more tractable diffusion approximation below. Furthermore, when 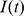 is not a homogeneous Poisson processes, closed form approximations can be obtained for both the deterministic and stochastic models.

Assume that 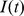 is a homogeneous Poisson process with rate 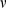. Then the increment, 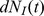, is independent from the current value of 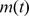 so that, by taking expectations in Eq. (8), 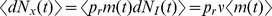 for the deterministic model. Similarly, 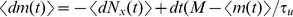. Combining these gives
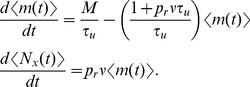
(9)Eq. (9) is also obtained by taking expectations in Eq. (7), which implies that the deterministic model and the stochastic model yield the same means when 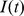 is a homogeneous Poisson process. The following equation for 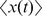 can be obtained using Eq. (9) and the fact that 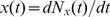,
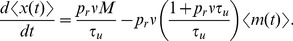
(10)


The stationary mean of 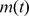 is given by the unique steady state solution to Eq. (9) [4],
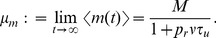
(11)Furthermore, after a perturbation of 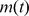 or starting from an initial condition 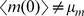, 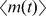 decays exponentially back to 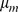 with time constant
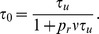
The stationary mean number of vesicles released by each presynaptic spike is given by 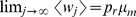 and the stationary mean of the postsynaptic signal is 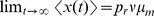, which represents the steady state rate of vesicle release. Furthermore, 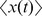 approaches its steady state exponentially with the same time constant, 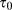, as 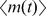.

The calculations of first moments above depend on the fact that 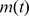 and 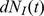 are independent for any 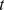. This can only be assumed to hold when Eq. (8) is interpreted in the It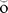 sense (so that 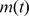 is updated directly after a spike) and 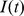 is a homogeneous Poisson process. If 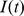 is not a homogeneous Poisson process, then the equations for the first moments are not valid and the first moments may not agree for the two models.

### A diffusion approximation of the stochastic model

Second moments for the stochastic model are difficult to derive analytically, so we obtain approximations by considering a diffusion approximation
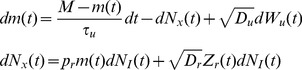
(12)where 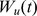 is a standard Wiener process that models stochasticity in vesicle recovery. Stochasticity in vesicle release is captured by the stationary process, 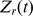, with moments given by 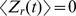, 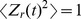, and 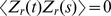 for 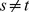. We assume that 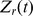, 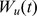, and 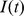 are mutually independent. These equations should be interpreted in the It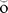 sense, so that the increments 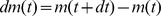 and 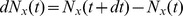 are independent from the history of the noise terms, 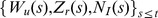, for any time 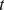 [49]. Since 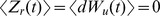, it is clear that the diffusion approximation defined by Eq. (12) has first moments that satisfy Eq. (9).

The noise coefficients, 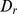 and 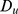, quantify the degree of randomness introduced by stochastic release and recovery respectively. To find appropriate values for these coefficients, we compute the infinitesimal variance of 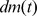 and 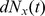 conditioned on the drift terms that appear in their respective equations in Eq. (12) [50]. Since vesicle recovery events are Poissonian, the variance of its increment is equal to its rate, giving the conditional variance
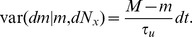
Note that the 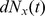 term that appears on the right hand side of Eq. (12) does not contribute to this conditional variance since 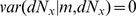. Conditioned on 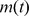 and the occurrence of a presynaptic spike, the number of vesicles released has a binomial distribution with mean 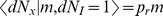 and therefore has conditional variance given by
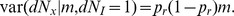
Optimally, we would set 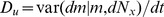 and 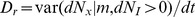, but doing so would give rise to nonlinear multiplicative noise in Eq. (12), which is difficult to treat mathematically. Instead, we obtain an approximation by replacing 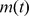 with its stationary mean, 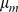, to obtain
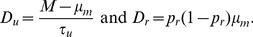
(13)All calculations for the stochastic model are carried out using the diffusion approximation from Eq. (12) with the noise coefficients from Eq. (13), and therefore expressions obtained are approximations to the full stochastic model described above. However, in all figures, simulations are performed using the full stochastic model from Eq. (7) (light blue lines) and show excellent agreement with the closed form approximations (dark blue lines).

Note that the deterministic model can be recovered by taking 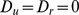 in Eq. (12). Thus, we can proceed in our analysis by considering Eq. (12) without instantiating 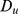 or 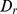 to obtain results that apply to both the deterministic and stochastic models.

### Derivation of the auto-covariance and power spectrum of 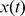


We quantify temporal and trial-to-trial variability between two stationary processes, 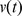 and 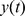, using the cross-covariance function,
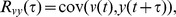
and its Fourier transform, the cross-spectrum,
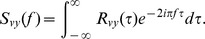
The cross-covariance (cross-spectrum) between a process and itself is called an auto-covariance (power spectrum). To quantify the variability of the postsynaptic response, we now derive the auto-covariance, 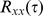, and the power spectrum, 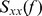, for the synapse model in Eq. (12).

From Eqs. (9) and (10) it is apparent that, for 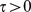, the expectations 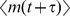 and 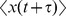 decay exponentially to their steady state, given any initial distribution, 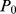, imposed on 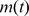 and 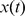. From this fact, it is apparent that 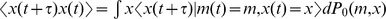 should inherit this exponential shape and therefore that 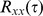 should have an exponential shape with time constant 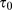.

We now make this argument more precise using a regression theorem from [49]. Define the bivariate Markov process,
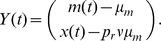
Then Eqs. (9) and (10) show that
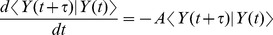
for 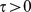 where
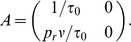
In Sec. 3.7.4 of [49], it is shown that this implies
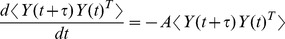
for 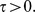 Solving this linear differential equation gives
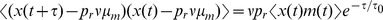
for 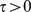. Thus, due to stationarity,
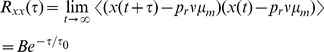
for 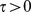 and where 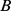 is a constant. By symmetry, we have 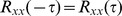. Note also that, since 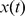 is a marked point process, there is a Dirac delta function that contributes to 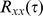 at 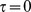 [51]. Finally, we may conclude that the auto-covariance of 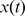 has the form
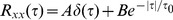
(14)for some constants 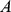 and 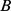.

To calculate the coefficients 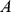 and 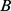 in Eq. (14), we must first calculate a few infinitesimal moments using stochastic calculus techniques [52]. In our calculations, we ignore terms of order 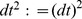 and higher, but must include terms of the form order 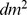 and 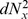 because their expectation is of the order 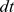 [50].

The second moment of 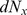 conditioned on 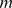 is given by
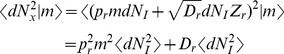
(15)

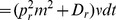
(16)where (15) follows from the fact that 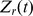 and 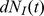 are independent from each other and from 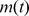, that 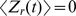, and that 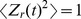; and (16) follows from the fact that 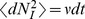. The calculation of the conditional mixed moment, 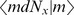, is similar and gives
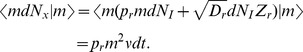



To calculate the stationary second moment, 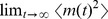, we modify a strategy from Sec. 4.4.7c of [49] to derive a linear differential equation for the time dependent second moment and find its steady state. First note that
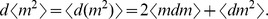
The first term in this sum is given by
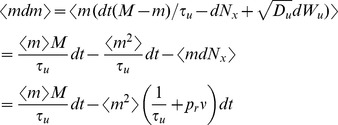
where we used the fact that 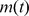 and 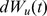 are independent (see above) and the last line follows from the equation for 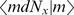 derived above. Now calculate
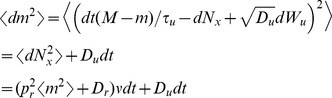
where we have eliminated terms of order 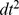 and used the fact that 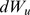 is independent from all other terms; and the last line follows from the equation for 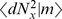 above. Combining these expressions gives a differential equation for the time course of the second moment of 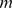,
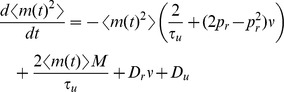
where 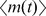 is given by the solution of Eq. (9) above. The stable fixed point of this linear differential equation is the stationary second moment of 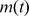,
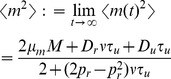
(17)where 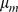 is the stationary mean of 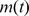, given in Eq. (11). The delta function in 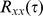 has area given by
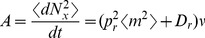
(18)where we used Eq. (16) above and where 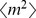 is given by Eq. (17).

To calculate the one-sided limit, 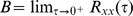, first calculate
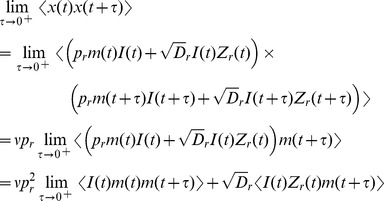
where we have used the fact that 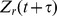 and 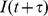 are independent of all of the other terms when 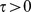. Each of the terms in the sum above can be calculated by conditioning on a spike at time 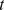 and on the value of 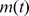,
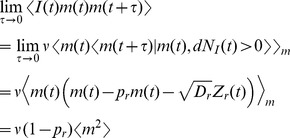
where 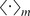 is expectation over the variable 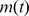. Similarly,
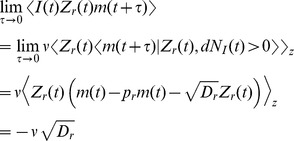
where 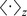 is expectation over 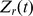. Combining the expressions above gives
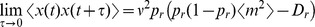
Finally, since 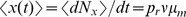 from above, we have
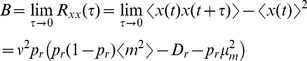
(19)where 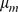 and 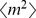 are the stationary first and second moments of 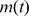, given in Eqs. (11) and (17). The auto-covariance of 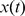 is then given by Eq. (14) with 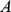 and 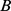 given by Eqs. (18) and (19).

The power spectrum is obtained from the auto-covariance through a Fourier transform,
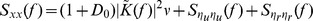
where
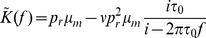
(20)is a deterministic linear kernel,
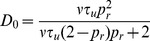
is the noise intensity introduced by the interaction between the stochastic input and deterministic vesicle dynamics,
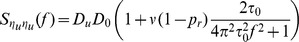
is the noise introduced by stochasticity in vesicle recovery, and
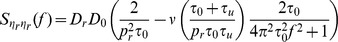
is the noise introduced by stochasticity in vesicle release. Note that 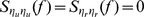 for the deterministic model since 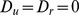.

### Derivation of the cross-covariance and cross-spectrum between 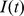 and 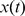


To measure the covariability between the presynaptic spike trains and the postsynaptic response, we now derive the cross-covariance between the input, 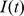, and the response 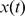. By a similar argument to the one made above for 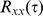, we may conclude that 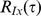 is the sum of a delta function and an exponential, except that the exponential is one-sided since 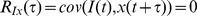 for 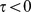. For 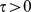, we can find the peak of the exponential by first conditioning on a spike at time 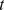, then conditioning on a spike at time 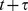,
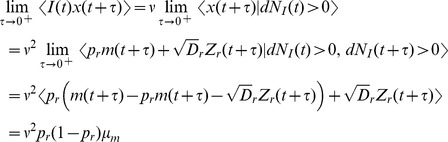
since 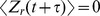. Thus,
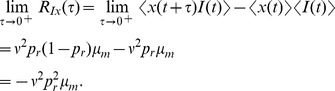
The area of the delta function in 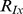 is given by
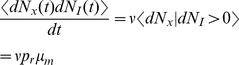
since 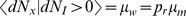. Thus, we have
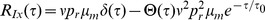
where 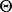 is the Heaviside step function. Taking the Fourier transform gives the cross-spectrum
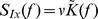
where 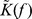 is defined in Eq. (20) above.

### Postsynaptic response to several correlated presynaptic spike trains

The statistics of the postsynaptic response to a population, 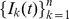, of *uncorrelated* presynaptic spike trains can be easily calculated from the statistics of individual responses, which are calculated above. However, neurons that contact a shared postsynaptic cell often exhibit correlations between their spiking activity [39], [53]. To determine the postsynaptic response to a population of correlated presynaptic spike trains, we must first calculate the pairwise cross-spectra of the conductances induced by these inputs. Assume that each spike train, 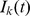, in the presynaptic population is a Poisson process with rate 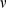. Introduce correlations by assuming that each pair, 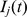 and 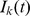, of spike trains share a proportion 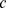 of their spike times so that 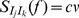 [54]. We use subscripts to denote quantities associated with each spike train and double subscripts as necessary. For simplicity, assume that the synaptic parameters 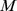, 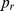, and 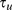 are identical for all synapses. The asymmetric case can be treated identically, but the expressions obtained are more cumbersome. The power spectrum, 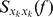, and the cross-spectrum, 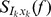, are given above (where they are written as 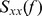 and 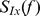). Below, we derive expressions for 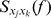 and 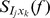 for 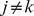.

First, following the same arguments used above to derive the moments of 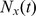 and 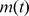 in the case of a single presynaptic spike train, we obtain the bivariate moments
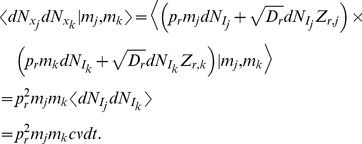
Similarly,
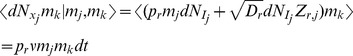
and, equivalently,
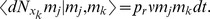
We now derive a differential equation for 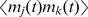 to get the stationary second moment. First note that 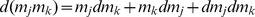 so that
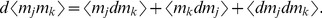
(21)By symmetry, the first and second terms in Eq. (21) are the same and they can be derived from Eq. (12) as
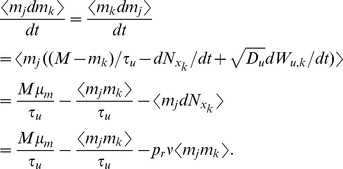
The last term in Eq. (21) is given by
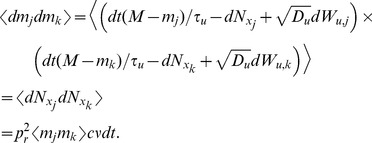
Combining these gives
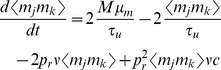
which has a fixed point at
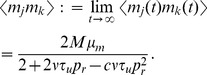
(22)


We now calculate the cross-covariance between 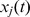 and 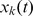. By a similar argument to that used to derive Eq. (14) above, the cross-covariance between 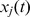 and 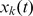 has the form
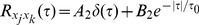
(23)where we have used the symmetry of 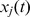 and 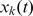, inherited from the symmetry in parameters, to conclude that 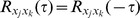. The area of the delta function is given by
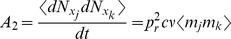
where 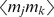 is given in Eq. (22). To find 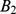, we first calculate
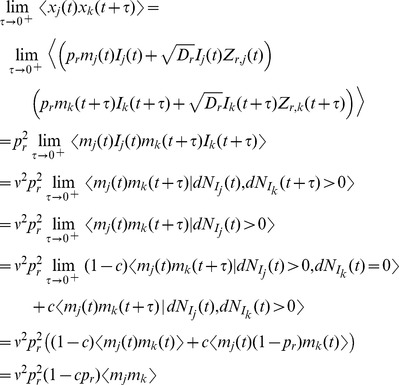
so that
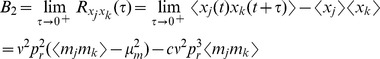
which gives 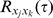 through Eqs. (22) and (23).

Finally, we will derive 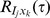 and 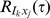. Once again, by linearity, each of these is the sum of a delta function and an exponential. The area of the delta function is given by
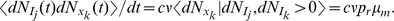
We also have
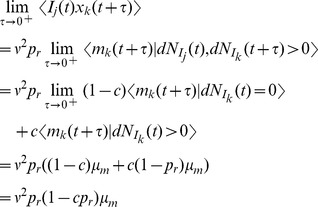
Thus,
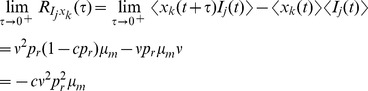
and therefore
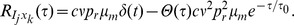
By symmetry, 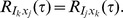


Finally, the cross-spectra can now be found through a Fourier transform to obtain
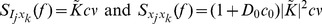
where
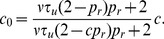
(24)


### Statistics of the postsynaptic conductance

So far we have described the statistics of the processes, 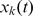, which quantify the release of vesicles released over time. The postsynaptic conductance induced by vesicle release is then defined as 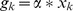 where 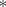 denotes convolution and 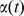 represents the time course of conductance induced by the release of a single vesicle (with 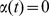 for 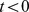). The statistics of 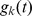 can easily be derived from those of 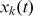 using standard signal processing identities [29] to give
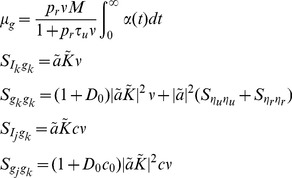
(25)for 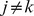 and the steady state variance of 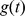 is given by 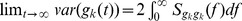.

### Synaptic filtering of presynaptic spike trains with rate coded signals

So far, we have discussed statistics of the conductance induced by a population of homogeneous Poisson presynaptic spike trains, but spike trains measured *in vivo* do not always exhibit homogeneous Poisson statistics [55]. For example, time-varying stimuli can induce fluctuations in the firing rate of presynaptic neurons. As a simple model of rate-coded signals, we assume that a shared, time-varying signal, 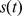, is encoded in the firing rates of a presynaptic population, 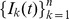.

In this model, each presynaptic spike train is a doubly stochastic Poisson process [51]. The instantaneous firing rate of each presynaptic neuron, conditioned on 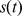, is given by 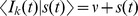. Without loss of generality, we assume that the signal has zero bias, 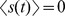, so that the unconditioned firing rates are 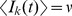. Signal correlations are introduced in this model by the shared signal, 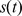. We include noise correlations, i.e., correlations that are not due to shared signal [38], [39], by assuming each pair of presynaptic spike trains share a proportion 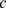 of their spike times.

To compute the auto- and cross-covariance functions we first note that, for 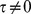,
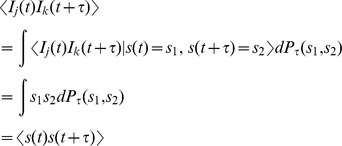
where 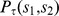 is distribution of 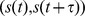 in the steady state (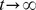). In addition, 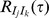 has a Dirac delta function at 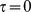 with mass equal to the rate of synchronous spikes, 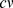. Thus, 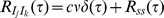 for 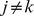. The auto-covariance (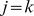) can be obtained by taking 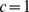. The cross-covariance function between 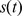 and 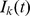 is be computed similarly to obtain 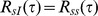. Taking Fourier transforms gives the spectra,
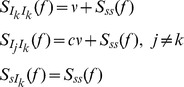
where 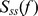 is the power spectrum of the signal.

Exact expressions for the statistics of the postsynaptic conductance are difficult to obtain for this inhomogeneous Poisson model because 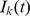 is correlated with 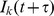 and with 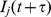, which invalidates the methods used in the derivations for the homogeneous Poisson model above. However, when 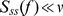, the firing rate inhomogeneities are weak compared to the background firing rate and temporal correlations are weak as a result (analogously, 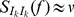). In this case, a linear approximation to the synaptic response can be obtained. To obtain this approximation, we find a linear filter that maps presynaptic spike trains to conductances and that is consistent with Eqs. (25) when inputs are Poisson. The following filter satisfies this requirement
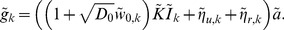
(26)Here, 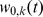 is standard Gaussian white noise, 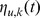 is unbiased stationary noise with power spectrum 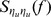 that accounts for stochasticity in vesicle recovery, and similarly for 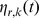, which accounts for stochastic vesicle release. The noise terms 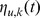 and 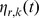 are zero for the deterministic model. All noise terms here are independent except that 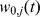 and 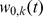 are correlated with cross-spectrum
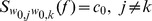
where 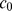 is given by Eq. (24).

The spectra predicted by Eq. (26) can be easily calculated using the fact that 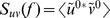 for stationary processes, 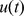 and 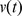, where 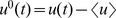 and 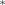 denotes complex conjugation [56]. Thus,
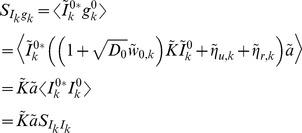
where we used the independence of the noise sources to eliminate several terms. Other spectra can be derived in a similar manner to obtain the following generalizations of Eqs. (25)
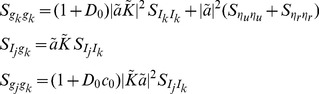
(27)for 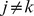. These expressions agree with Eqs. (25) when inputs are Poisson, i.e., when 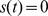, because 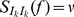 and 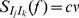 in this case. When 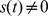 and 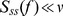, these expressions give a linear approximation which is verified using simulations in several figures below. The fidelity with which the signal, 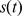, is represented in the conductances, 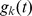, depends on the cross-spectrum which can be calculated in analogous manner to 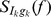 above to obtain
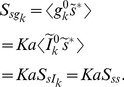
(28)


We are especially interested in the population spectra, 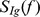, 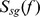 and 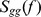, where 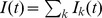 is the total presynaptic input and 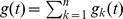 is the total conductance induced by 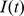. These are given by using the bilinearity of covariances to obtain
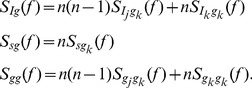
(29)


A similar inhomogeneous Poisson input model was used in [17] to investigate the transfer of rate-coded signals for the deterministic model of synaptic depression. Their model is analogous to our deterministic model with 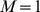 (since their response amplitudes are normalized) and 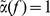 (since they consider the postsynaptic response, 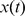, before convolution with a conductance kernel). Under these substitutions, our expression for 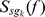 agrees with their expression for 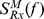 exactly (where we use an “
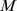
” superscript to indicate expressions from [17]). However, our expression for 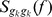 for the deterministic model only agrees with their expression for 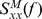 when 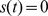 (i.e., when the input is a homogeneous Poisson process). Our expression has an additional term that accounts for power introduced by the signal 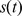. In particular, 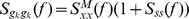 for the deterministic model when 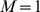 and 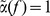.

### Parameters used for figures

Theoretical results are obtained for arbitrary parameter values, but for all figures we use the parameters from Table 1, which are chosen to represent values from experimental studies. The values used for 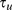 and 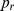 have been deemed “typical” for pyramidal-to-pyramidal synapses in the rodent neocortex [2], [19] and the value of 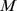 is typical for several cortical areas [34]. The form of 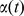 is chosen to model AMPA dynamics and its units are rescaled so that 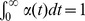. This rescaling simplifies the exposition in the Results.
